# Ontogenetic changes in larval swimming and orientation of pre-competent sea urchin *Arbacia punctulata* in turbulence

**DOI:** 10.1242/jeb.129502

**Published:** 2016-05-01

**Authors:** Jeanette D. Wheeler, Kit Yu Karen Chan, Erik J. Anderson, Lauren S. Mullineaux

**Affiliations:** 1 Biology Department, Woods Hole Oceanographic Institution, Woods Hole, MA 02543, USA; 2 Division of Life Science, Hong Kong University of Science and Technology, Clear Water Bay, Kowloon, Hong Kong; 3 Applied Ocean Physics and Engineering Department, Woods Hole Oceanographic Institution, Woods Hole, MA 02543, USA; 4 Department of Mechanical Engineering, Grove City College, Grove City, PA 16127, USA

**Keywords:** Pluteus, Behavior, Hydrodynamics, Particle image velocimetry

## Abstract

Many marine organisms have complex life histories, having sessile adults and relying on the planktonic larvae for dispersal. Larvae swim and disperse in a complex fluid environment and the effect of ambient flow on larval behavior could in turn impact their survival and transport. However, to date, most studies on larvae–flow interactions have focused on competent larvae near settlement. We examined the importance of flow on early larval stages by studying how local flow and ontogeny influence swimming behavior in pre-competent larval sea urchins, *Arbacia punctulata*. We exposed larval urchins to grid-stirred turbulence and recorded their behavior at two stages (4- and 6-armed plutei) in three turbulence regimes. Using particle image velocimetry to quantify and subtract local flow, we tested the hypothesis that larvae respond to turbulence by increasing swimming speed, and that the increase varies with ontogeny. Swimming speed increased with turbulence for both 4- and 6-armed larvae, but their responses differed in terms of vertical swimming velocity. 4-Armed larvae swam most strongly upward in the unforced flow regime, while 6-armed larvae swam most strongly upward in weakly forced flow. Increased turbulence intensity also decreased the relative time that larvae spent in their typical upright orientation. 6-Armed larvae were tilted more frequently in turbulence compared with 4-armed larvae. This observation suggests that as larvae increase in size and add pairs of arms, they are more likely to be passively re-oriented by moving water, rather than being stabilized (by mechanisms associated with increased mass), potentially leading to differential transport. The positive relationship between swimming speed and larval orientation angle suggests that there was also an active response to tilting in turbulence. Our results highlight the importance of turbulence to planktonic larvae, not just during settlement but also in earlier stages through morphology–flow interactions.

## Abstract (precis)

**Highlighted Article:** Pre-competent, 6-armed larval urchins swim faster and are less stable in experimental turbulent flow than younger 4-armed larvae, suggesting a potential age/morphology-driven differential transport mechanism in ambient flow conditions.

## INTRODUCTION

Many marine invertebrates have complex life histories in which the planktonic larval phase acts as the vehicle to connect otherwise disjointed benthic adult populations which are mostly non-mobile (Cowen and Sponaugle, 2009; Levin, 2006). Larval supply, in terms of both the number of settlers and their condition, plays an important role in determining population dynamics and community interactions (Pechenik, 2006; Pineda et al., 2007). Larval swimming behaviors in response to various chemical, biological and physical cues have important implications for the adult populations (Metaxas and Saunders, 2009). For planktonic, pre-competent larvae, swimming behaviors significantly impact vertical distribution patterns, which in turn shape dispersal (Clay and Grünbaum, 2010; Sameoto et al., 2010). For older, competent larvae, behaviors around settlement sites could significantly affect recruitment patterns (Hadfield and Koehl, 2004; Wethey, 1986; Zimmer and Butman, 2000).

One such set of behavior-triggering cues are the hydromechanical signals associated with turbulent flow that larvae experience in nature (Eckman, 1996; Roy et al., 2012). These hydromechanical signals can be interpreted as three interacting components, namely acceleration, rotation and deformation (Vogel, 1994). Larvae may respond to combinations of these signals in an active and/or passive manner. In the water column, strong swimming larvae such as crab zoea actively change their swimming speeds in response to turbulence intensity (Welch and Forward, 2001) and barnacle cyprids swim upwards to counteract downwelling currents (DiBacco et al., 2011). For moderate swimming larvae such as oyster larvae, a plasticity in response to turbulence has been observed, where competent-to-settle larvae have been observed to both sink (Fuchs et al., 2013) and swim upward (Fuchs et al., 2015; Wheeler et al., 2013) in high turbulence; these active behavioral responses may be regulated by body size. For weaker swimming plankton, such as larval sand dollars, it has been observed that their morphologies primarily interact passively with ambient flow (Chan, 2012; Clay and Grünbaum, 2010). Plutei of larval sand dollars represent an ‘armed morphology’, for which organisms use ciliated extensions (such as arms or lobes) for feeding and swimming. This type of dual-use structure is shared between many weakly swimming marine invertebrate larvae, such as other echinoderms, mollusks and some polychaete larvae (Strathmann and Grünbaum, 2006). Shuttlecock-like pluteus larvae have long ciliated arms supported by a calcite skeleton (Pennington and Strathmann, 1990), and add pairs of arms (from 4-armed, to 6-armed, and to 8-armed) through their development. Plutei are characterized by fore–aft asymmetry (Chan, 2012) and, in some species, a distinct density differential in which mass is concentrated in the posterior (Mogami et al., 2001). Both these morphological characteristics of pluteus larvae induce passive reorientation in low Reynolds number conditions, by two different mechanisms. Fore–aft asymmetry coupled with negative buoyancy produces a gravity-induced hydrodynamic torque (Roberts, 1970), while a differential density produces a torque proportional to the distance between the larva's center of gravity and center of buoyancy (Mogami et al., 2001; Roberts, 1970). Both mechanisms likely play a role in the observed reorientation of larvae in flow, but previous experimental evidence suggests that urchin larvae primarily rely on non-homogeneous density, or ‘bottom heaviness’ to reorient (Mogami et al., 2001). The larvae–turbulence interaction is therefore a combination of both active behavioral choices such as those of barnacle cyprids and larval oysters and passive biomechanical limitations imposed by the pluteus morphologies of larval sand dollars and sea urchins.

The three-way interactions between larval behavior, larval morphology and the surrounding flow have significant implications for population distribution. Clay and Grünbaum (2010) reported that 4- and 8-armed larval sand dollars are more likely to be passively reoriented into downwelling regions by tilting in shear flow. This tilting in shear flow suggests an inability to maintain a stable orientation (hereafter stability) – the larva's usual vertically directed swimming with arms in an upright position. Such ontogenetic differences in stability could imply distinct passive mechanisms to mediate transport and vertical position through ontogeny, and, indeed, field observations of larval sand dollars suggest that older larvae are more likely to be found in deeper waters (Pennington and Emlet, 1986). Swimming–flow interactions could also affect distribution patterns through aggregation. Some weakly swimming plankton, e.g. *Heterosigma akashiwo*, exhibit strong spatial and temporal patchiness driven by gyrotactic motility in shear: directed motion resulting from the orientation of cell axes through the balance of viscous and gravitational torque (De Lillo et al., 2014; Durham et al., 2013; Durham and Stocker, 2012). Therefore, quantifying swimming behaviors of planktonic larvae in environmentally relevant flow fields is essential for understanding transport and dispersal.

To date, however, most studies have focused on larvae–turbulence interactions of competent larvae preparing to settle (but see Chan et al., 2015; McDonald, 2012; Roy et al., 2012). Various studies highlight that larvae actively respond to turbulence or components of turbulence, e.g. larval boat snails *Crepidula fornicata* increase upward swimming with increasing turbulence level (Fuchs et al., 2010), larval sea slugs *Phestilla sibogae* retract their vela when encountering turbulent filaments containing chemical cues (Hadfield and Koehl, 2004), and larval eastern oysters *Crassostrea virginica* dive when experiencing high fluid acceleration over short time intervals (Wheeler et al., 2015). Together with other modeling studies, these earlier works suggest turbulence enhances larval settlement (Eckman, 1990; Fuchs et al., 2015; but see Pearce et al., 1998, who suggested that an increase in turbulence reduces settlement in scallop larvae). Recently, deformation associated with horizontal shear has been suggested to induce competency in larval urchins (Gaylord et al., 2013) and to induce cloning in coral larvae (Heyward and Negri, 2012). While there is increasing information about how late-stage larvae respond to realistic flow fields near settlement, there is still relatively little understanding of how weakly swimming ciliated planktonic larvae respond to turbulence in earlier life stages and how that response may change through ontogeny.

In this study, we exposed the weakly swimming planktonic larvae of the sea urchin *Arbacia punctulata* to three environmentally relevant turbulent flow regimes at two different developmental stages (4- and 6-armed plutei). We hypothesized that (1) larvae actively modify their swimming speeds in response to ambient flow conditions such that older, larger larvae swim faster and (2) larvae are passively reoriented in flow such that older, larger larvae are more stable as a result of bottom heaviness. Using non-invasive video tracking and flow subtraction techniques, we investigated the effect of turbulence on swimming speed and stability of larval *A. punctulata* through ontogeny.

## MATERIALS AND METHODS

### Study organism and larval culturing

The study organism is the purple urchin *A. punctulata* (Lamarck 1816), which has long been a focus species of embryonic and larval development studies (Harvey, 1932; Hinegardner, 1969) with well-established eco-toxicological responses (Nacci et al., 2000). Two male and two female *A. punctulata* adults were procured from the Marine Biological Laboratory Animal Supply (Woods Hole, MA, USA) and were injected with 0.55 mol l^−1^ KCl to induce spawning (Strathmann, 1987). Oocytes collected were washed through a 63 µm mesh to remove debris and sperm were collected dry. Eggs of each female were fertilized in 0.22 µm-filtered seawater (∼18°C, 32 psu) with sperm solutions from both males at concentrations of approximately 1000 sperm ml^−1^. Fertilization success was visually confirmed by the presence of a fertilization envelope in >95% of the eggs at 20 min post-fertilization. Maternal cultures were reared separately and later combined in equal proportions for the experimental observations.

At 24 h post-fertilization, hatched embryos from each female were transferred into six 16 l plastic containers (12 containers in total) holding filtered seawater (0.22 µm filtered, 32 psu) at a density of ∼6 individuals ml^−1^. This rearing density was chosen to provide a sufficient number of larvae for subsequent video observations. These containers were aerated and kept in an environmental chamber maintained at 18±1°C. Larvae were fed *ad libitum* with a combination of *Isochrysis galbana* and *Dunaliella tertiolecta* at 15,000 and 2500 cells ml^−1^, respectively, daily. Complete water changes were performed every 3 days. Daily 20 ml subsamples were taken from each container and examined under a microscope to confirm normal larval development.

### Video observations of larval swimming in turbulence

Swimming behaviors of larvae in different levels of turbulence were observed at 8 and 23 days post-fertilization (4- and 6-armed larval stages, respectively). These observations were made in a Plexiglas tank (44.5×44.5×90 cm) equipped with two vertically oscillating grids identical to that detailed in Wheeler et al. (2013). Larval swimming and fluid motion were observed in a vertical cross-section field of view (FOV) of 3.5×3.5 cm in the center of the tank using a monochrome high-speed video camera (Photron Fastcam SA3) at 60 frames s^−1^. The FOV was illuminated with a sheet of light from a near-infrared laser (Firefly 300 W, 1000 Hz, 808 nm; Oxford Lasers) set perpendicular to the optical axis of the camera.

Four replicate trials were conducted for each stage using different batches of larvae, with the tank drained and rinsed between trials. Larvae were subsampled from all culturing vessels, i.e. larvae from both mothers were simultaneously used for these experimental trials. Larval urchins were gently poured into the tank from a beaker, with approximately 60,000 and 28,000 larvae introduced per trial at day 8 and day 23, respectively, for larval densities of <0.5 individuals ml^−1^. Neutrally buoyant polystyrene particles of 3–3.4 µm diameter (Spherotech, 2.5 cm^3^ of a 10% w/v suspension) were injected into the tank as passive tracer particles for particle image velocimetry (PIV).

During each trial, larval urchins were exposed to three different treatment levels by oscillating the grids at 0, 0.25 and 0.5 Hz at a fixed amplitude of 5 cm. The energy dissipation rates of these three regimes were previously computed using a separate set of PIV experiments and the resultant 2D flow vectors as per Doron et al. (2001), and were estimated to be ∼0, 0.002 and 0.017 cm^2^ s^−3^ (Wheeler et al., 2013). Kolmogorov length scales for the two higher flow regimes were 0.147 and 0.088 cm, respectively, and the integral length scales were 3.023 and 3.649 cm, respectively, corresponding to the ranges of the smallest to dominant energy-containing eddies in the tank (Wheeler et al., 2015). The mean 4- and 6-armed larval midline lengths, for comparison, were approximately 0.013 and 0.018 cm, respectively. Hereafter, these three turbulence treatments are referred to as unforced, low forcing and moderate forcing. The qualitative terms are intended to describe the relative intensity of the turbulence in the range of what larvae might experience in field conditions. While surf zone conditions can reach energy dissipation rates of 10^0^ to 10^4^ cm^2^ s^−3^ (Gaylord et al., 2013), tidal and estuarine flows are calmer (10^−2^ to 10^0^ cm^2^ s^−3^), with energy dissipation rates decreasing further away from the coast (Gross and Nowell, 1985). Our turbulence forcing regimes reflect energy dissipation rates of the open ocean and of calmer nearshore waters. Larvae were exposed to increasing turbulence from unforced flow to moderate forcing in a sequential manner. Five minutes of spin up time at the beginning of each turbulence level allowed the flow to equilibrate before filming began, and multiple 45 s video clips (5–11 clips) were collected at each turbulence level. Video clips were collected until an adequate total number of larvae (>100) had been observed in the FOV. In between treatments of low and moderate forcing, the grids were stopped to allow flows to dissipate. Additional unforced flow video clips were collected during this interval after transient net downward flow was no longer visible in the FOV. All video clips were exported and saved as high-resolution (1024×1024 pixels) TIFF images and used for larval tracking and flow visualization.

**Fig. 1.**
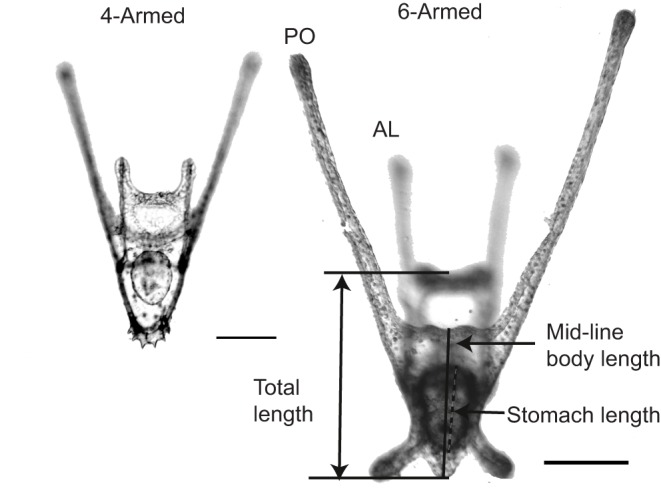
**A comparison of the relative size and morphology of the 4- and 6-armed larva.** Scale bars (lower right), 100 μm. PO, postoral arm; AL, anterolateral arm. Reported length scales are stomach length, mid-line body length and total length (where total length is defined as the distance from the base of the body to the oral hood, neglecting arm length).

At the end of each trial, larval urchins were collected on a 100 µm mesh and fixed in buffered 2% paraformaldehyde for later microscopy and image analysis. Body lengths, lengths of skeletal arm rods and stomach were measured using the software Fiji (Schindelin et al., 2012; Fig. 1).

### Larval tracking

Observed larval movement is a combination of both individual swimming motion and advection by surrounding fluid, so we adapted the flow subtraction method from Wheeler et al. (2013, 2015) to test whether swimming behaviors alone vary with changes in flow conditions. To compute larval velocities in such a relative framework (isolated from advection in flow), we first identified larval positions in each frame and calculated absolute velocity of individuals in the flow field. We subsequently estimated local flow velocities using PIV and subtracted these to isolate larval swimming velocities from advection.

**Fig. 2.**
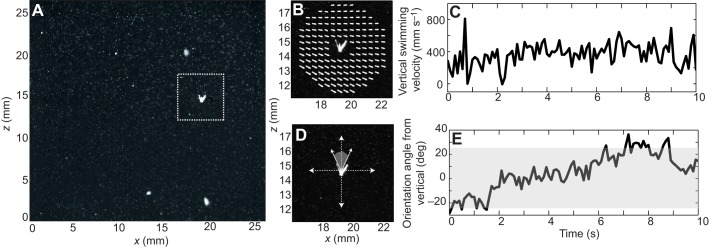
**Larval movement and orientation tracking.** (A) Sample experimental image, where an in-focus larva (with arms clearly visible) is highlighted by the white dashed box. Smaller white specks are passive particles and larger diffuse spots are out of focus larvae. (B) Close-up of the highlighted larva, with overlaid particle image velocimetry (PIV) velocity field (white arrows) surrounding it. Passive particle intensity is dimmed for clarity. (C) Sample time series of an individual larva's vertical swimming velocity as it was tracked in the field of view. (D) Close-up of the highlighted larva, with overlaid Cartesian coordinate system and shaded range of angles (−25 to 25 deg from vertical) at which the larva was considered upright. (E) Sample time series of an individual larva's orientation angle from vertical as it was tracked in the field of view. The shaded region denotes the range of orientation angles at which the larva was considered upright.

The flow velocities were calculated using LaVision DaVis imaging software (v7.9). Each high-resolution TIFF image (for example, Fig. 2A) was subdivided into 16×16 pixel interrogation windows with no overlap and the average displacement of particles in each window between images was determined by a multi-pass 2D fast Fourier transform (FFT) analysis scheme. Velocity fields were converted into MATLAB data files for use in the subsequent flow subtraction, and smoothed with a 10 time step interval boxcar filter to eradicate high-frequency noise. Full details of the PIV procedure are presented in Wheeler et al. (2013).

To track individual larvae, the TIFF images were filtered to remove background noise and thresholded for brightness, leaving larvae to appear as white silhouettes on a black background. The centroid position of individual larvae was recorded frame by frame using customized LabView 2010 (National Instruments) software. Larval tracks were then compiled with in-house MATLAB (v7.12.0 R2011a) software by connecting centroids frame-by-frame within a user-defined threshold distance, and used to calculate larval velocity in the FOV.

### Flow subtraction and larval velocity computation

Larval positional data were integrated with PIV flow field data in MATLAB, where an annulus of flow velocity vectors around each larva was identified in each frame (Fig. 2B). The annulus had an inner radius equal to the sum of the larval radius and the PIV spatial grid interval, to mask poor PIV velocity estimates near the larval position. The outer radius was 4 times the inner radius, to incorporate ∼4–6 velocity vectors away from the larva in all directions. The flow velocity at the larval position was then estimated: a second-order two-dimensional Taylor expansion was fitted to the annulus velocity vectors and interpolated to the larval position. Larval swimming velocities (independent of advection in the flow) were calculated at each time step (Fig. 2C) by subtracting the flow velocity (interpolated to the larval position) from the absolute velocity. For each larva, we denote swimming speed at each time step as **V**_s_=[*u*_s_, *w*_s_], where **V**_s_ is overall swimming velocity vector, *V*_s_ is the vector magnitude, or speed, and *u*_s_, *w*_s_ are horizontal and vertical velocities, respectively.

### Larval orientation

To identify larval orientation, TIFF images were imported into MATLAB and subsampled in time to 10 frames s^−1^. A subset of larvae was randomly selected (per video clip), and from larval position data these larvae were magnified individually to facilitate the identification of the larval orientation axis. The larval orientation axis was determined by extending the body midline to a point equidistant between the visible pair of arms (anterolateral or posterodorsal), and this process was repeated frame by frame to construct a time series for the orientation angle of each larva. A total of 16 larval trajectories (sampled at 10 frames s^−1^) in each turbulence regime (3 levels) for each of the 4 trials were analyzed in this way, for a total of 192 larval trajectories in each of the 4- and 6-armed groups. We computed the angular deviation between orientation axis and the true Cartesian vertical (defined here as 0 deg, using the FOV as the reference), hereafter referred to as larval orientation angle (Fig. 2D). Using the orientation time series of each larva (Fig. 2E), we computed the proportion of time spent upright by normalizing the duration in which the orientation angle lies between ±25 deg from the vertical with the total time the larva was tracked. The identification of orientation axes was not automated because of the highly variable appearance of the multi-armed larvae in two dimensions. Attempts to automate orientation computations led to heavy biases toward the angles of the arms that were closest to the focal plane, which were much brighter than the more distant arms. While the range of angles from vertical defining an upright orientation (±25 deg) was chosen somewhat arbitrarily, analysis with various magnitudes of deviation showed that our subsequent statistical conclusion is not overly sensitive to this parameter.

### Statistical analysis

We compared changes in larval size between 4- and 6-armed stages using a *t*-test. We tested for the effect of turbulence level on vertical and horizontal swimming velocities, swimming speed and stability using non-parametric statistical methods because the dataset did not meet the assumption of equal variance. We compared the swimming speeds (*V*_s_) and velocities (*u*_s_, *w*_s_) across all forcing regimes between the two stages with a Mann–Whitney test. We also compared the effect of the turbulence regime on the distributions of horizontal velocity (*u*_s_), vertical velocity (*w*_s_) and speed (*V*_s_) within the 4- and 6-armed stages separately with Kruskal–Wallis tests. We compared the effect of turbulence regime on stability at each of the two developmental stages using the proportion of time spent upright as a metric with Kruskal–Wallis tests, and identified possible directional biases in orientation angle with a Wilcoxon signed-rank test. We further explored the relationship between orientation angle and horizontal velocity (*u*_s_), vertical velocity (*w*_s_) and swimming speed (*V*_s_) with a Spearman rank correlation for each of the developmental stages.

## RESULTS

### Larval size and swimming speed increased with ontogeny

As larval urchins developed, their overall size significantly increased, with an average mid-line body length of 131.0±10.1 µm at the 4-armed stage and 175.8±15.5 µm at the 6-armed stage (*t*-test, *F*=135.1, *P*<0.001; Table S1). Considering all turbulence regimes combined, swimming speed (*V*_s_) increased through ontogeny, with an average flow-subtracted swimming speed of 748.6±14.6 µm s^−1^ at the 4-armed stage and 953.5±18.9 µm s^−1^ at the 6-armed stage (*U*=1,921,191, *P*<0.0001). However, vertical and horizontal velocities varied with stage in a different pattern, such that there was no significant effect of stage on horizontal velocity but a significant effect on vertical velocity (*U*=1,212,659, *P*<0.0001).

### Swimming speeds changed with turbulence level but differed between stages

**Fig. 3.**
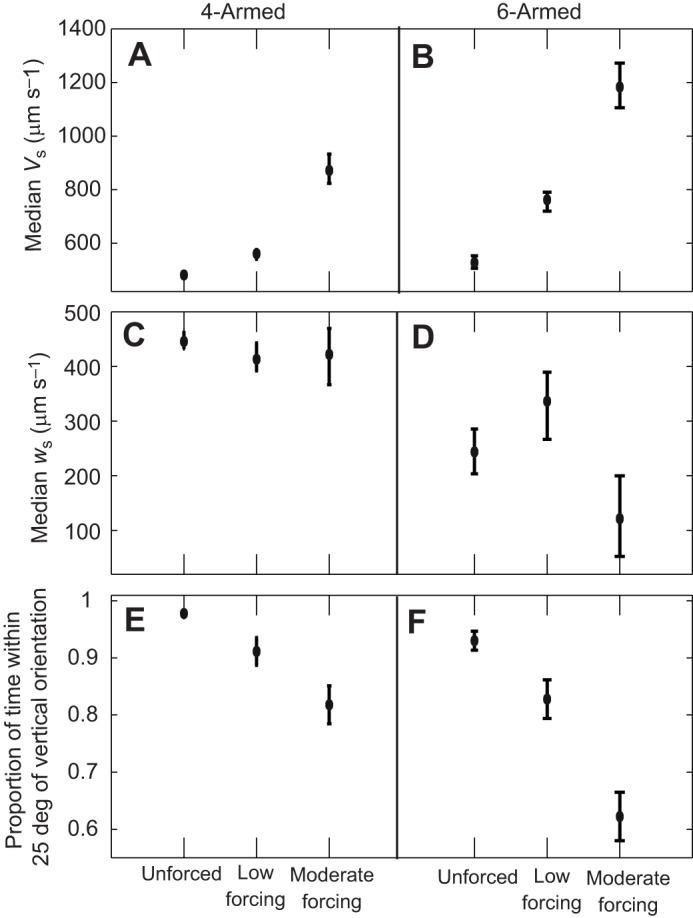
**Larval swimming speed and time spent in vertical orientation at different turbulence intensities.** (A,B) Median larval swimming speed (*V*_s_) with 95% confidence intervals, with respect to the turbulence regime, for 4-armed larvae (A) and 6-armed larvae (B). (C,D) Median larval vertical swimming velocity (*w*_s_) with 95% confidence intervals, with respect to the turbulence regime, for 4-armed larvae (C) and 6-armed larvae (D). (E,F) Mean proportion of time spent by larvae within ±25 deg of the vertical orientation with 95% confidence intervals, with respect to the turbulence regime for 4-armed larvae (E) and 6-armed larvae (F).

Generally, larval urchins swam in relatively straight, upwardly directed paths. Their swimming speeds varied significantly between turbulence regimes at both 4-armed (*K*=438.8, *P*<0.001; Fig. 3A) and 6-armed stages (*K*=542.1, *P*<0.001; Fig. 3B), where overall speed (*V*_s_) increased monotonically with increasing turbulence intensity for both stages. The turbulence regime had a significant effect on vertical velocity (*w*_s_) for both 4-armed (*K*=6.446, *P*=0.040; Fig. 3C) and 6-armed stages (*K*=21.763, *P*<0.001; Fig. 3D). However, the two developmental stages demonstrated distinct relationships between turbulence and vertical swimming velocity: at the 4-armed stage, vertical velocity decreased slightly with increasing turbulence (Fig. 3C), such that vertical velocity was fastest in the unforced regime with a median vertical velocity of 445 µm s^−1^; at the 6-armed stage, vertical velocity increased and then decreased with increasing turbulence (Fig. 3D), such that vertical velocity was highest in the low forcing regime with a median vertical velocity of 337 µm s^−1^, compared with 244 µm s^−1^ in the unforced regime and 122 µm s^−1^ at moderate forcing. Larval horizontal velocities (*u*_s_), however, did not significantly change with turbulence at either the 4-armed (*K*=4.896, *P*=0.086) or 6-armed stage (*K*=0.784, *P*=0.676).

### Stability decreased with turbulence and age

Larval orientation angle in all observed cases averaged close to vertical (0.9±16.5 deg), with >73% of all observations within ±10 deg. The median angle did not significantly differ from zero (*W*=−472, *P*=0.914), demonstrating that the larvae as a population had no preferred direction (positive or negative) in orientation angle from vertical. As expected, turbulence did not bias larval orientation angle, as the flow was near isotropic and homogeneous. The distribution of the median angle was unimodal, demonstrating that there were not multiple groups of larvae with preferred directional angles that averaged zero. However, transient tilting occurred frequently in turbulence. Larvae at both developmental stages spent over 95% of the time in the upright position in the unforced regime (Fig. 3E,F), but time spent upright decreased significantly with turbulence for both 4-armed (*K*=17.33, *P*<0.001; Fig. 3E) and 6-armed larvae (*K*=27.62, *P*<0.001; Fig. 3F). Older larvae appeared more susceptible to tilting than younger larvae: in the moderate forcing regime, the younger 4-armed larvae were upright on average 87.6±2.9% (±s.e.) of the observed time compared to 69.7±4.1% (±s.e.) at the 6-armed stage.

### Swimming speed correlated with orientation angle

**Fig. 4.**
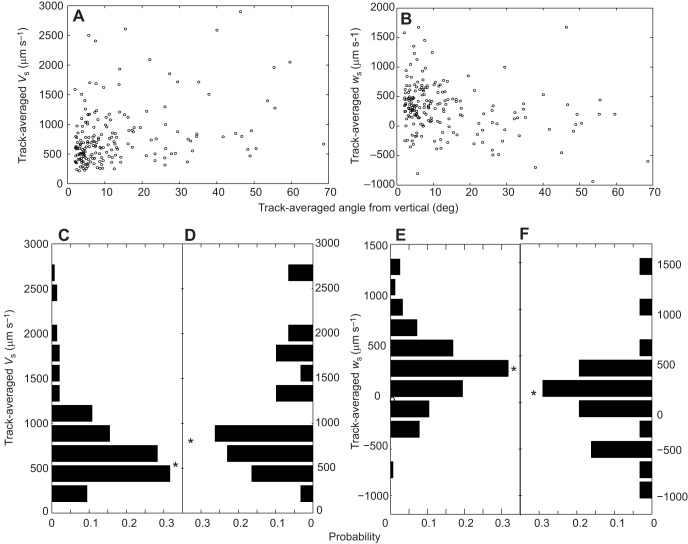
**Correlation between swimming speed and orientation angle.** (A,B) Track-averaged orientation angle (0 deg is vertical) versus track-averaged swimming speed (*V*_s_; A) and track-averaged vertical swimming velocity (*w*_s_; B). Each point represents an individual 6-armed larva, using larvae from all turbulence regimes. (C–F) Probability distribution of track-averaged swimming speed in upright larvae (within ±25 deg of vertical; C) and tilted larvae (outside ±25 deg of vertical; D), and probability distribution of track-averaged vertical swimming velocity in upright larvae (E) and tilted larvae (F). Asterisks denote median speed or velocity for each distribution. Note that the *x*-axes for the tilted larvae (D and F) are reversed, so that they are mirror images of C and E, respectively.

We explored the relationship between larval orientation (measured from the vertical, 0 deg) and swimming speed across all flow regimes and found a considerable difference between the 4- and 6-armed stages. At the 4-armed stage, there was no significant relationship between the orientation angle and overall swimming speed (*V*_s_), horizontal velocity (*u*_s_) or vertical velocity (*w*_s_) (*P*>0.126). In contrast, at the 6-armed stage, the slight deviations from vertical orientation (Fig. 4A, data points at lower left) were significantly correlated with increased swimming speed (Spearman rank ρ=0.163, *P*=0.002; Fig. 4A). There was no significant correlation between horizontal velocity and orientation angle (Spearman rank ρ=0.0716, *P*=0.325). Vertical velocity, however, was negatively correlated with orientation angle (ρ=−0.221, *P*=0.002; Fig. 4B), i.e. tilted larvae were more likely to sink than upright larvae. The sinking of larvae as a result of negative buoyancy explains how it is possible to have negative vertical velocities even though orientation angles are less than 90 deg (Fig. 4B). Observed variance in swimming speed is due in part to the range in body size of observed larvae (in addition to behavioral components) and the variance may partially obscure the changes in larval behavior when tilted. Probability distributions of swimming speed (Fig. 4C,D) and vertical velocity (Fig. 4E,F) for upright versus tilted larvae highlight the changes in behavior: median swimming speed in upright larvae was 624.1±34.8 µm s^−1^ (Fig. 4C) and increased to 817.3±116.5 µm s^−1^ in tilted larvae (Fig. 4D). Upright larvae had a median vertical velocity of 346.5±31.3 µm s^−1^ (Fig. 4E), while tilted larvae had a median vertical velocity of 91.2±87.6 µm s^−1^ (Fig. 4F).

## DISCUSSION

Marine invertebrate larvae swim in a complex fluid environment, and their responses to hydromechanical signals during their planktonic and near-settlement stages have significant implications for transport, survival and recruitment. By exposing two different stages of larval urchin *A. punctulata* to different turbulence regimes in the laboratory, we found that larval swimming speed increased and vertical velocity changed with increasing turbulence. Vertical velocity of the older 6-armed larvae increased at low forcing but decreased when they experienced moderate forcing, suggesting more downward movement. This is consistent with our observations that 6-armed larvae were more unstable when exposed to turbulence and spent less time in the upright position compared with 4-armed larvae. Such changes in stability through ontogeny can potentially lead to changes in depth distributions as larvae age: larvae may occupy wider or narrower bands of depth, or shift from surface to deeper water. Such changes to vertical distributions may in turn impact larval survival and transport.

The swimming speed and vertical velocity of larval urchins changed significantly with age and turbulence regime (Fig. 3). The older, larger, 6-armed larval urchins had higher swimming speeds than the 4-armed larvae, and this increase is consistent with earlier hydromechanical model predictions that weight-carrying capacity is proportional to total arm length (Grünbaum and Strathmann, 2003). Here, weight-carrying capacity is defined as the maximum net downward force, i.e. the difference between gravitational pull and buoyancy sustained by a larva at zero velocity. However, this increase in swimming speed with turbulence did not translate to an increase in vertical velocity. For 4-armed larvae, the median vertical velocity of the population was smaller in both the forced regimes compared with the unforced regime. For 6-armed larvae, median vertical velocity presented a dome-shaped response with increasing turbulence, such that vertical velocity was largest at low forcing and smallest at moderate forcing. Contrary to findings in other marine invertebrate larvae, e.g. mollusk veligers (Fuchs and DiBacco, 2011; Wheeler et al., 2013), the vertical velocity of larval urchins did not increase with increased turbulence. However, these results are consistent with previous observations of other echinoderms through ontogeny: older larval sand dollars and larval green urchins have a lower vertical velocity under no flow or shear conditions compared with younger larvae (Chan et al., 2015, 2011).

The observed differences in vertical velocity between 4- and 6-armed larvae may stem from a changing morphology as they age. While the increased number and length of arms could provide more weight-carrying capacity (Grünbaum and Strathmann, 2003), the associated increase in the calcite skeleton structure could potentially counteract this increase in weight-carrying capacity and incur metabolic costs for upward swimming while carrying additional weight (Pennington and Strathmann, 1990). The importance of this second factor is supported by our observations of lower vertical swimming velocities in 6-armed larvae when compared with 4-armed larvae, across all turbulence regimes. However, as the functional response of vertical swimming velocity to turbulence regime also differed between the two developmental stages, we conclude that the ambient flow must also play a role, and that observed changes in swimming velocity are not solely driven by the balance between weight-carrying capacity and size. Some potential explanations for the between-stage differences are structural: the additional pair of posterodorsal arms in the 6-armed larvae have fenestrated skeletal rods, providing drag to reduce passive sinking speeds (Emlet, 1983), and these arms are connected to the larval body by muscles that allow larvae to orient these arms to redirect local flow fields or permit reverse swimming (Lacalli and Gilmour, 1990). The ability to increase drag and redirect flow might help 6-armed larval urchins to maintain to a certain degree their vertical velocities at low forcing, in comparison to unforced or moderate forcing regimes, where they may not engage these strategies.

Like swimming velocity, larval orientation also changed significantly with age and turbulence regime. Larvae of both age groups spent significantly less time upright as turbulence intensity increased, and 4-armed larvae were significantly more stable than 6-armed larvae in all turbulence regimes. Ontogenetic changes in larval shape may play a role in the larval ability to maintain an upright orientation and, hence, stability in flow. Older, heavier larval urchins have increased skeleton weight as ballast, which could enhance stability (Grünbaum and Strathmann, 2003; Mogami et al., 2001). However, the addition of arms and increased size could also compromise stability by affecting the separation distance between the center of gravity and center of buoyancy (Grünbaum and Strathmann, 2003). Individuals with a longer or wider spread of arms might also more readily cross stream lines, thus experiencing a larger fluid torque (Chan, 2012; Clay and Grünbaum, 2010). These biomechanical constraints may help account for the observation that 6-armed larvae were more prone to tilting in higher turbulence compared with smaller 4-armed larvae.

Our results highlight the importance of morphology in creating biomechanical constraints for movement. The pluteus morphology of echinoids varies significantly between families, but different morphological structures could serve a similar purpose: for example, the arbaciids studied here have posterolateral arms; spatangoids have both posterolateral arms and an apical process (De Amaral et al., 2007). In both cases, these extensions could potentially act to counterbalance larvae in moving water. Computational fluid dynamics modeling work on functional morphology and associated tradeoffs of ‘armed’ larvae would significantly contribute to our understanding of the ecology and evolution of larval forms (Chan, 2012; Kiørboe et al., 2014; Miller et al., 2012).

Our observations suggest that pre-competent larval urchins respond to turbulence with both active and passive mechanisms: they actively increase their swimming speed in increased turbulence and are passively reoriented through morphology–flow interactions, which compromise their ability to maintain directed swimming. When exposed to increased turbulence, 6-armed larvae had higher swimming speeds but lower vertical swimming velocities than 4-armed larvae. It is important to note that a decrease in such time-averaged vertical velocity in the 6-armed larvae does not necessarily represent a reduction in movement with turbulence. Instead, this change may reflect an increase in the proportion of time spent swimming in directions other than the preferred vertical orientation, suggesting a passive response to turbulence. This is supported by the significant negative correlation between orientation angle and vertical swimming velocity. However, the observed increase in swimming speed in higher turbulence regimes demonstrates that larvae are not merely being reoriented and persisting in a default swimming mode: the positive correlation between orientation angle and swimming speed demonstrates that larvae swim faster when they are tilted, or potentially that stronger swimmers are more prone to tilting.

The first interpretation, that tilted larvae swim faster, suggests an intriguing possibility that an active behavior is triggered by a passive larval response to local flow conditions. While tilting and reorientation are likely passive responses to increased shear in turbulence, an increased swimming speed subsequent to tilting is an active response. Larval responses to ambient flow may involve more such interactions between active and passive responses than have previously been investigated. Despite the absence of a statocyst-like structure in larval urchins, our results support early observations that they have mechanoreception ability and are capable of adjusting their propulsive behaviors accordingly (Mogami et al., 2001, 1988; Wada et al., 1997). Further work on ciliary motion control (Gustafson et al., 1972; Shiba et al., 2002) and gene expression (e.g. orthologs of vertebrate mechanosensory genes, such as Sp-Usherin and TRPA1; Sodergren et al., 2006; M. Volnoukhin, Mechanosensory cells and swimming behaviour of embryos of the sea urchin *Strongylocentrotus purpuratus*, MSc thesis, Simon Fraser University, 2010) could help us to understand the biophysical mechanisms of behavioral control under natural flow conditions.

The second interpretation, that faster swimming larvae are more prone to tilting, also has a sound biomechanical basis. Gallager (1993) demonstrated using tethered swimming shipworm larvae that the flows generated by swimming larvae decay more rapidly away from the larval body as swimming speed increases. Self-induced flows may shield larvae from background turbulence, and so slower swimming may confer greater stability. Future investigations of larval swimming in moderate and high turbulence regimes where tilting is most prevalent will help to further elucidate the relationship between orientation angle and swimming speed, and to determine larval response times for an active swimming response to tilting.

The observed changes in response to turbulence intensity through larval development have significant implications for larval distribution. If our observations in the laboratory can translate to the water column in the coastal ocean, older, 6-armed larvae would be more likely to be tilted, have slower vertical velocity, and move into downwelling regions to be downwardly transported in these energetic environments (Clay and Grünbaum, 2010). This differential swimming could result in selective transport, leading to ontogenetic differences in depth distribution (Chan et al., 2011; Grünbaum and Strathmann, 2003). One possible scenario is that the differential transport helps younger individuals to maintain themselves in the surface water where food abundance is higher and help older individuals to approach their settlement sites. Alternatively, differences in response and distribution could reflect different strategies to avoid predation. One possible hypothesis is that smaller, 4-armed larvae are less vulnerable to predators relying on vision and, hence, can survive even in well-illuminated surface waters. Our results suggest that the observation of early stage pre-competent larvae in realistic flow conditions, in addition to competent larvae seeking settlement sites, is important for understanding the population dynamics of marine invertebrates. Our observations also demonstrated that a gyrotaxis-like reorientation response to turbulence is present in multicellular swimming plankton. Turbulence, therefore, not only impacts larval transport but also could potentially shape the aggregation and distribution of other zooplankton and thereby impact ecological interactions through the modulation of patch dynamics.
